# Timing of Muscle Response to a Sudden Leg Perturbation: Comparison between Adolescents and Adults with Down Syndrome

**DOI:** 10.1371/journal.pone.0081053

**Published:** 2013-11-20

**Authors:** Maria Stella Valle, Matteo Cioni, Mariangela Pisasale, Maria Rosita Pantò, Antonino Casabona

**Affiliations:** 1 Department of Bio-Medical Sciences, Section of Physiology, University of Catania, Catania, Italy; 2 Gait Analysis Laboratory, Physical Medicine and Rehabilitation Residency Program, University of Catania, Catania, Italy; 3 Department of Clinical and Molecular Bio-Medicine, University of Catania, Catania, Italy; 4 Italian Down People Association, section of Catania, Catania, Italy; The University of Queensland, Australia

## Abstract

Movement disturbances associated with Down syndrome reduce mechanical stability, worsening the execution of important tasks such as walking and upright standing. To compensate these deficits, persons with Down syndrome increase joint stability modulating the level of activation of single muscles or producing an agonist-antagonist co-activation. Such activations are also observed when a relaxed, extended leg is suddenly released and left to oscillate passively under the influence of gravity (Wartenberg test). In this case, the Rectus femoris of adults with Down syndrome displayed peaks of activation after the onset of the first leg flexion. With the aim to verify if these muscular reactions were acquired during the development time and to find evidences useful to give them a functional explanation, we used the Wartenberg test to compare the knee joint kinematics and the surface electromyography of the Rectus femoris and Biceps femoris caput longus between adolescents and adults with Down syndrome. During the first leg flexion, adolescents and adults showed single Rectus femoris activations while, a restricted number of participants exhibited agonist-antagonist co-activations. However, regardless the pattern of activation, adults initiated the muscle activity significantly later than adolescents. Although most of the mechanical parameters and the total movement variability were similar in the two groups, the onset of the Rectus femoris activation was well correlated with the time of the minimum acceleration variability. Thus, in adolescents the maximum mechanical stability occurred short after the onset of the leg fall, while adults reached their best joint stability late during the first flexion. These results suggest that between the adolescence and adulthood, persons with Down syndrome explore a temporal window to select an appropriate timing of muscle activation to overcome their inherent mechanical instability.

## Introduction

Down syndrome (DS) is a common genetic condition associated with a number of cognitive and movement disturbances. Morphometric analysis by means of magnetic resonance images or autopsy reports have revealed many neurological abnormalities associated with cognitive, social and emotional disorders [1]. Problems in the field of motor control can be ascribed mainly to the smaller cerebellum that is considered the source of the decreased muscle tone and the disturbances in postural and movement coordination [2]. In addition to hypotonia distributed to all major muscle groups, the movement difficulties also depend on biomechanical defects such as the ligamentous laxity [3], flat feet [4] and obesity [5]. Because of these deficits, people with DS constantly challenge the joint instability increasing the risk to impair motion and posture in everyday life. However, despite their inherent neuronal and musculoskeletal disorders, persons with DS express large capacity of adaptation, implementing compensatory strategies to overcome their deficits [6]–[10].

In a previous paper we evaluated the mechanical properties of the knee joint of adults with DS by means of the Wartenberg test [11], a procedure applied to assess passive knee motion [12]–[14]. During the test, the subjects were required to relax as the leg was suddenly released from an extended position and left to oscillate passively about the gravitational resting point. We expected to observe a constant muscle tonic activity throughout the test, but the Rectus femoris (RF) produced bursts of activation shortly after the onset of the first flexion, restoring a low tonic activity over the following oscillations. Most of the bursts exhibited latencies typical of long-loop responses that are considered associated with the activity of cortical and subcortical structures [15]–[17]. Thus, we argued that the adults with DS use complex and flexible neural circuits to adaptively increase the joint stability in response to unexpected perturbations.

The possibility exists that these reactions are not innate but they might emerge, over developmental time, from simpler and less flexible behaviors. The development of children with DS is delayed, achieving basic motor tasks, such as walking and upright standing, about 1 year later with respect to typical development subjects [18], [19]. Some authors indicated that during the preadolescence, persons with DS exhibit the highest possibility to select resources useful for a better movement and posture stabilization [20], [21]. Considering the slowness of development and the potentiality of modifications observed in teenagers with DS, we predicted that a comparison between adolescents and adults might reveal differences in muscle reactions during the execution of the Wartenberg test. In particular, our expectation was that adolescents would exhibit both short- and long-latency activations as a sign of a selection process, whereas in adults would prevail long-latency responses. In order to verify this schema and collect evidences for a functional interpretation, we compared the kinematics of the knee motion and the EMG activity of RF and of Biceps femoris caput longus (BFcl) of a group of adolescents with respect to a sample of adults with DS.

## Materials and Methods

### Ethics statement

All subjects gave informed consent according to the Declaration of Helsinki. A written informed consent was obtained by parents or guardians of all participants and the experimental procedures were approved by the ethics committee of the University of Catania.

### Subjects

Forty-five persons with DS (25 adults and 20 adolescents) from the Italian Down People Association were recruited to participate in this study. Eligibility criteria for inclusion were as follows: good general health, understanding of simple verbal instructions, no history of seizures, no current medications, no previous surgical procedures on the lower extremities. The exclusion criteria comprised the inability to relax and a low compliance to the test. According to these criteria, 15 adults and 10 adolescents were excluded from the experiments and 10 young adults (six men and four women; age: 25.5±3.7 yrs) and 10 adolescents (four boys and six girls; age: 13.9±3.1 yrs) were selected (Table 1). We resampled the group of adults with respect to our previous paper [11]. The two youngest subjects included in the group of adolescents in table 1 (subject #4 and #6) were at the boundary between childhood and adolescence (10 and 9 yrs respectively), but considering that, for the purpose of this study, some level of variability can be acceptable, we left these girls in the group and for brevity we adopted the term “adolescents” throughout the paper. We did not include parallel groups of persons without DS since our main goal was to detect muscle activations typically absent during the Wartenberg test in both adults and children without DS [11], [13], [22].

**Table 1:** Age, gender and anthropometric data of participants.

Adolescents	Gender	Age	Height	Weight	Leg-foot	Adults	Gender	Age	Height	Weight	Leg-foot
#1	f	12	144	50	38	#1	m	26	154	62	43
#2	m	17	158	74	43	#2	m	22	147	70	42
#3	f	11	137	40	35	#3	m	29	145	71	41
#4	f	10	119	30	34	#4	m	25	152	60	44
#5	f	17	140	51	42	#5	f	30	152	60	44
#6	f	9	120	26	35	#6	m	21	158	61	45
#7	f	16	143	51	34	#7	f	27	147	60	40
#8	m	16	153	65	43	#8	m	21	151	50	40
#9	m	14	153	63	42	#9	f	31	148	64	43
#10	m	17	152	49	44	#10	f	23	141	65	37

Height (cm), Weight (kg), L-F length  =  Leg-Foot length (cm).

### Procedures

**Figure 1:**
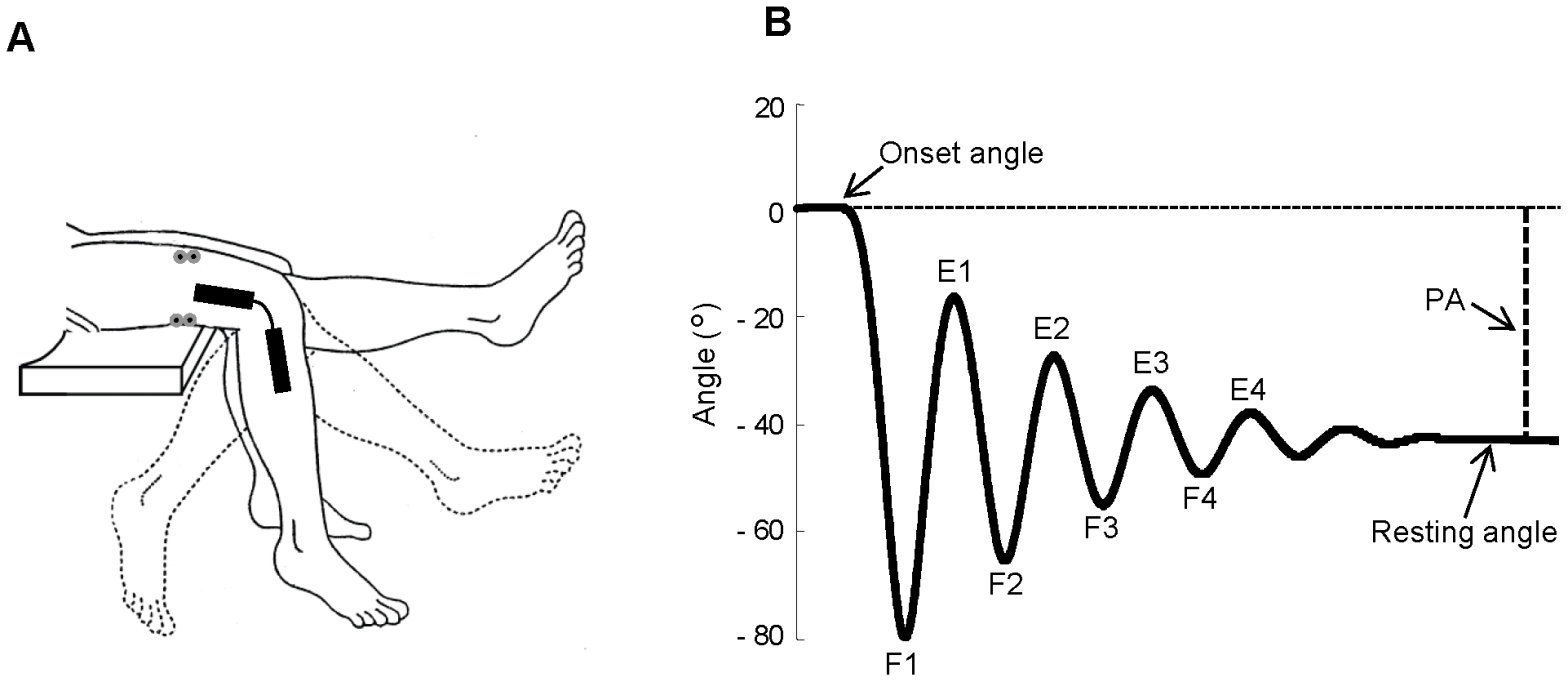
Experimental set up to perform Wartenberg test. (A) From the maximum extension of knee joint, the leg passively oscillates about the gravitational resting position. An electrogoniometer acquired the knee joint angle and two couples of surface electrodes recorded the EMG activity from the Rectus femoris and Biceps femoris caput longus. (B) Typical kinematic trajectory reporting the knee angle variations from the onset angle to the resting angle, and the main landmarks for further kinematic and dynamic computations. F1–F4, angles of reversal at the end of each swing flexion; E1–E4, angles of reversal at the end of each swing extension; PA, plateau amplitude.

Each subject was examined in the sitting up position with the trunk inclined approximated 40° from the horizontal to provide a comfortable starting position. For each trial, the examiner first positioned and supported the leg at the maximum extension, then, when the subject was relaxed, she unexpectedly and quickly removed her support allowing the leg to fall and swing passively between flexions and extensions until it stopped at the resting position (Fig. 1A). Participants received extensive explanation and demonstration of the procedure and were asked to relax for the entire duration of each trial; we placed a special care to ensure that all the persons had full understood the instructions. The Wartenberg test was performed on the right limb and repeated 10 times for each subject with 1 min of rest between the trials. The same examiner carried out the test across all the sessions.

### Recording systems

Angular displacements of the knee joint motion were recorded by an electrogoniometer (Biometrics Ltd, Gwent, UK) placed on the lateral side of the limb with the two arms aligning with the thigh and leg axes (Fig. 1A). Kinematic data were low-pass filtered with a zero-lag second-order Butterworth filter with 6 Hz cutoff frequency. Angular trajectories were monitored on-line and trials showing abnormal or erratic oscillations were rejected.

EMG data were collected using surface electrodes placed over the RF and BFcl. EMG signals were preamplified at the electrode site and sampled at 1 kHz. The EMG activity was first high-pass filtered at 20 Hz to remove movement artifacts, and then it was full wave rectified for further signal conditioning. Since the electrodes position over the BFcl made uncomfortable the experimental session for six adults and seven adolescents, in these subjects the EMG activity was recorded only for the RF. All the measurements were sampled at 1 kHz and acquired by a portable device integrating EMG and electrogoniometer recordings (PocketEMG by Bioengineering Technology and System, BTS, Milan, Italy). Kinematic data were resampled at 200 Hz for further processing.

### Measurements and estimations

Kinematic and EMG measurements were elaborated to obtain the following parameters (Fig. 1B): onset angle, corresponding to the angle at leg maximum extension before the onset of the first flexion; resting angle, corresponding to the angle at rest position; plateau amplitude, measured between onset angle and resting angle; angles of reversal at the end of each swing flexion (F1–F4) and extension (E1–E4); relaxation index  =  F1/PA, that is the normalized first peak flexion angle; mean value and peak angular velocity of knee movements during the first flexion; mean value and first and second peak angular acceleration during the first flexion; EMG area, computed over the interval of the first flexion by the integral of the zero-phase low-pass filtered EMG signal (100 Hz, 2 pass, 2^nd^ order Butterworth); latency of EMG responses.

For the latency measurements, the onset of the leg movement was agreed on by two of the authors after visual testing if the trace of the velocity profile was greater than its baseline in successive 25 ms windows. The onset of EMG bursts was determined using the nonlinear Teager–Kaiser energy operator (TKEO). Although in our experiments clear bursts of phasic activity were recorded, for the EMG traces we preferred to adopt a procedure able to achieve a robust onset detection performance. Compared to other methods that consider only the signal amplitude in determining the onset time, TKEO takes into consideration both amplitude and frequency by computing the energy of the EMG signal. It has been demonstrated that this method improves the signal-to-noise ratio and increases the accuracy of the EMG onset detection [23], [24]. For each EMG trace, the TKEO was applied on the 20 Hz high pass filtered raw signal and the TKEO output was full wave rectified. The threshold T to identify the onset time over the TKEO domain was determined as follows: 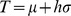
where *μ* and σ are the mean and standard deviation of a reference background chosen from 100 ms before the leg movement onset to the movement onset; *h* is a preset variable, defining the level of the threshold. We assigned to *h* the value of 7 according to the data from Li et al. [23] and after preliminary tests conducted on a sample of our EMG traces. The window for searching the value exceeding the preset threshold was comprised between the onset of the movement and the first peak flexion (F1).

To describe the patterns of muscle activations, we applied a Principal Components Analysis (PCA) to each of the two datasets of EMG activities. The EMG traces recorded in all trials and in all subjects were zero-phase low-pass filtered (10 Hz, 2 pass, 2^nd^ order Butterworth), normalized over the interval of the first flexion and, for each group, a small number of Principal Components (PCs) was extracted. The resulting PCs are uncorrelated waveforms representing the most common patterns across each of the two groups. The original elements of the datasets can be reconstructed from the linear sum of all the PCs weighted by coefficients that describe their relationships with the original EMG traces (weighting coefficients). These coefficients represent the Pearson product-moment correlation between the elements of the original dataset (EMG waveforms) and each PC computed. In order to simplify the interpretation of the PCs waveforms and their relationship with the EMG responses, we applied varimax rotation, which is an orthogonal rotation that changes iteratively the PCs to achieve maximal separation in the distribution of the response vectors in PCs space (for an extensive review on the PCA methodology see [25]).

All filtering in our analyses was run forward and reverse to eliminate time lag.

Finally, we used kinematic and anthropometric data to estimate the damping and stiffness coefficients for the first three flexions and extensions of knee joint. The computation of damping and stiffness coefficients was restricted to passive motion and based on a second order underdamped system that combine the linear elements of stiffness, damper, and inertia. See our previous paper for details of the computational procedure [11]. The values of damping and stiffness coefficients were normalized by the gravitational torque (GT) determined as follows: 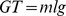
where, *m* represents the leg-foot complex mass; *l* represents the distance between the rotation axis of knee joint to the leg-foot complex's center of oscillation; *g* represents the gravitational acceleration. Anthropometric measurements of the body weight, body height and leg-foot length (see Table 1) were collected and used to predict mass characteristics (*m* and *l*) according to the anthropometric tables provided by Winter [26].

### Statistical analysis

Mean and standard deviation of each parameter were calculated pooling together all trials performed on each session by each subject. Comparisons between groups were performed by using Student's *t*-test.

Damping and stiffness coefficients over three cycles were modelled by a general linear model with repeated measures, using the consecutive cycles as within-subjects factor and the groups as between-subjects factor. Post hoc *t*-tests were computed for differences between the groups at each cycle. Linear regression models were performed to correlate EMG activity with mechanical parameters.The level of significance for all tests was set to p<0.05.

All analyses were performed using Matlab, version R2012a (Mathworks Inc, Natick, MA, USA) and SYSTAT, version 11 (Systat Inc., Evanston, IL, USA).

## Results

**Figure 2:**
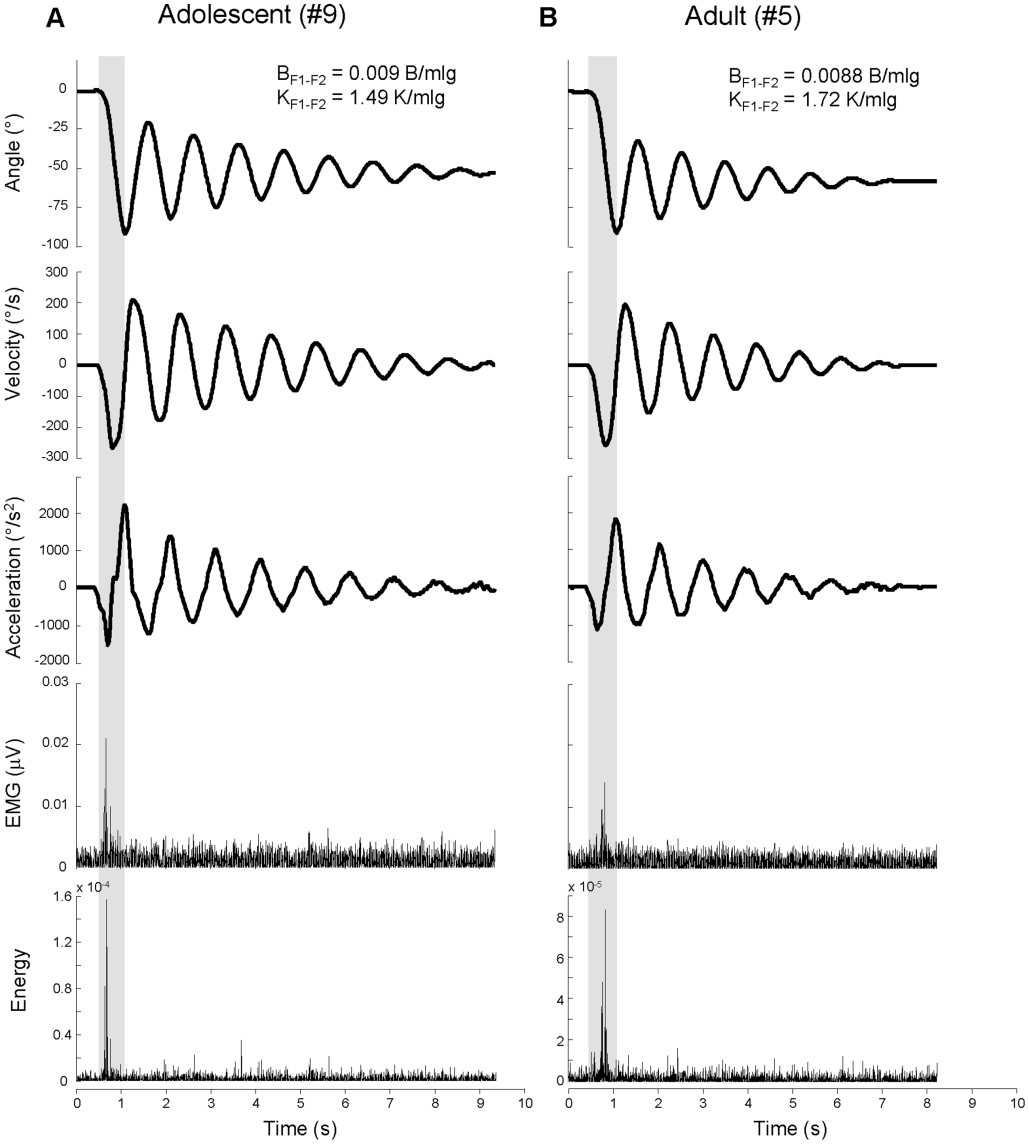
Examples of kinematic and EMG traces. Kinematic and EMG traces recorded in one adolescent (A) and one adult (B) with Down syndrome. Shadow areas highlight the excursion of the first flexion. B_F1-F2_ and K_F1-F2_ are normalized damping and stiffness coefficients for the first extension-flexion cycle after the first fall. The last two rows represent the full wave rectified EMG without (fourth row) and with (fifth row) TKEO signal condition.

All subjects showed a phasic muscular activity during the first flexion of the Wartenberg test, followed by relaxed free oscillations. Two examples from one adolescent and one adult are illustrated in figure 2A–B. Normalized values of damping and stiffness coefficients for the first extension-flexion cycle (B_F1-F2_ and K_F1-F2_, respectively) are reported in the upper part of figure 2A–B. The kinematic traces show that the adolescent compared with the adult exhibited larger peaks of acceleration during the first flexion (shadow areas) and smaller value of K_F1-F2_. These differences accounted for the longer duration of the oscillations in the adolescent than the adult. The values of B_F1-F2_ were similar in the two subjects. The EMG onset of the RF burst during the first flexion occurred at short time from the movement initiation for the adolescent (71 ms) and much later for the adult (270 ms). The onset times were detected from the EMG traces conditioned by TKEO procedures (bottom traces in Fig. 2).

### Mechanical data

**Figure 3:**
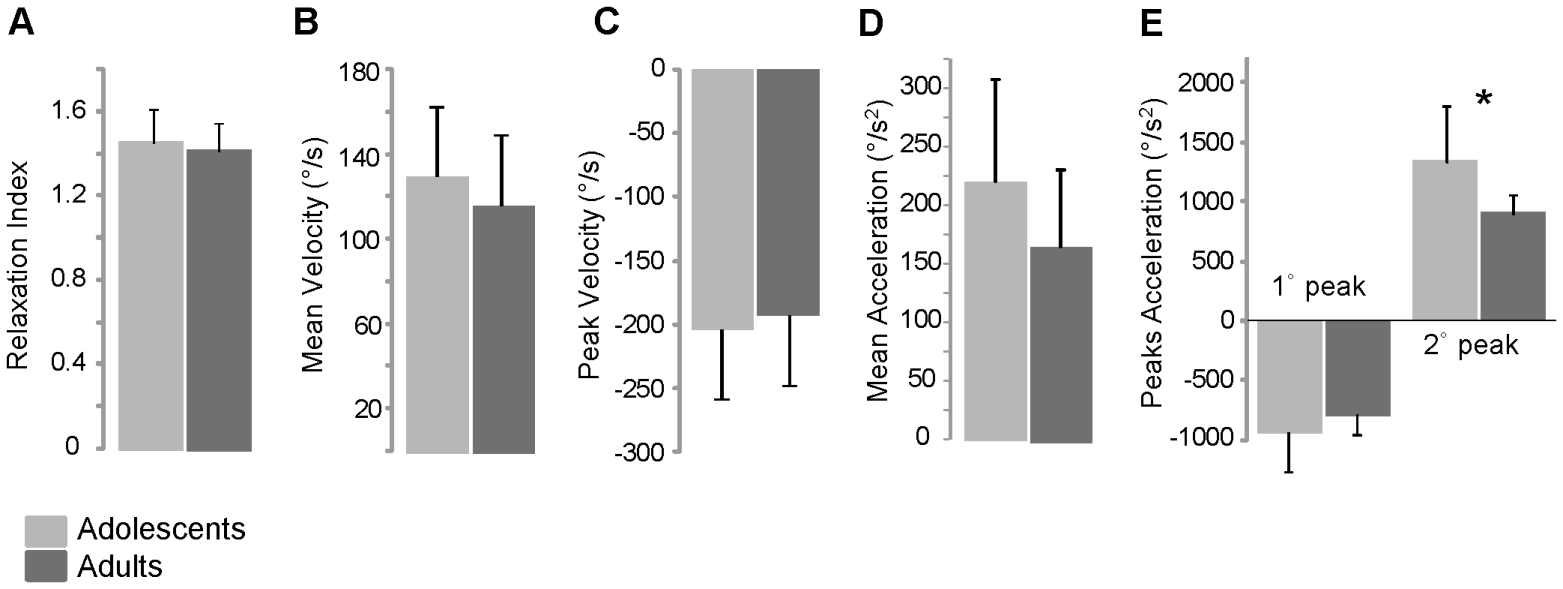
Summary of statistic of kinematic parameters. Means and standard deviations of kinematic parameters measured in adolescents and adults with Down syndrome (A–E). * P<0.05.

The summary of statistical analysis concerning the kinematics of the first flexion is reported in figure 3A–E. Adolescents and adults exhibited similar average values comparing relaxation index (Fig. 3A), mean and peak velocity (Fig. 3B–C) and mean and first peak acceleration (Fig. 3D–E); the only significant difference between the two groups was the second peak acceleration (p<0.05; Fig. 3E).

**Figure 4:**
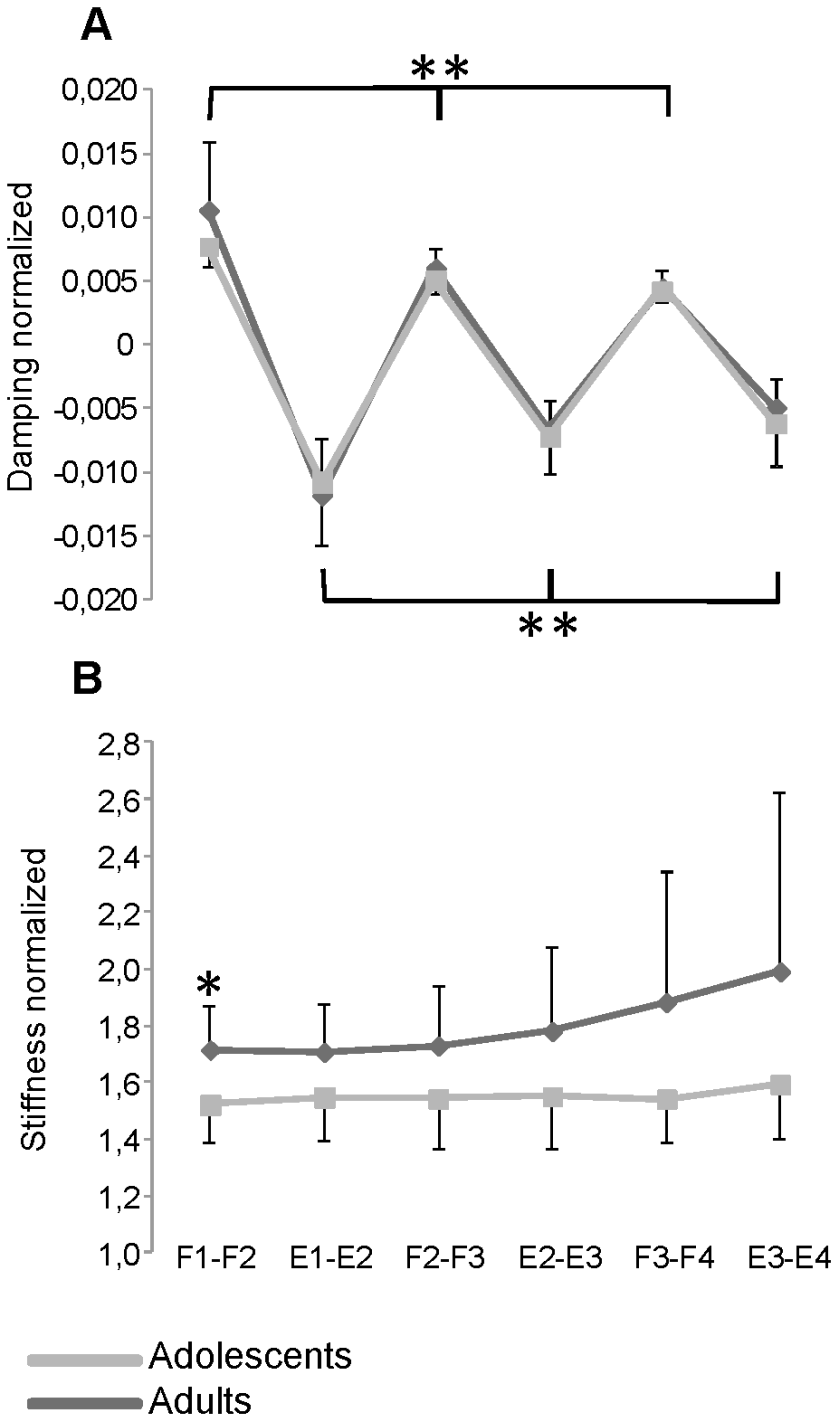
Damping and stiffness coefficients variations. Damping (A) and stiffness (B) coefficients estimated for adolescents (grey line) and adults (black line) with Down syndrome over the three cycles following the first flexion. F1–F4, peak angles of flexion; E1–E4, peak angles of extension. Data were normalized to the gravitational torque and expressed as means and standard deviations.* P<0.05. ** P<0.01.

The variations of the damping and stiffness coefficients across the three oscillations following the first flexion were almost all comparable between the two groups (Fig. 4A–B). However, the damping coefficient showed a typical effect of cycles, changing significantly throughout the three extension-flexion cycles (positive values in Fig. 4A; F_1,2_ = 12.17, p<0.01) and between the successive three flexion-extension cycles (negative values in Fig. 4A; F_1,2_ = 37.25, p<0.001). On the contrary, the stiffness coefficient was not statistical significant between cycles, but at the first extension-flexion cycle, adults exhibited higher stiffness than adolescents (p<0.05; Fig. 4B).

### Electromyographic data

**Figure 5:**
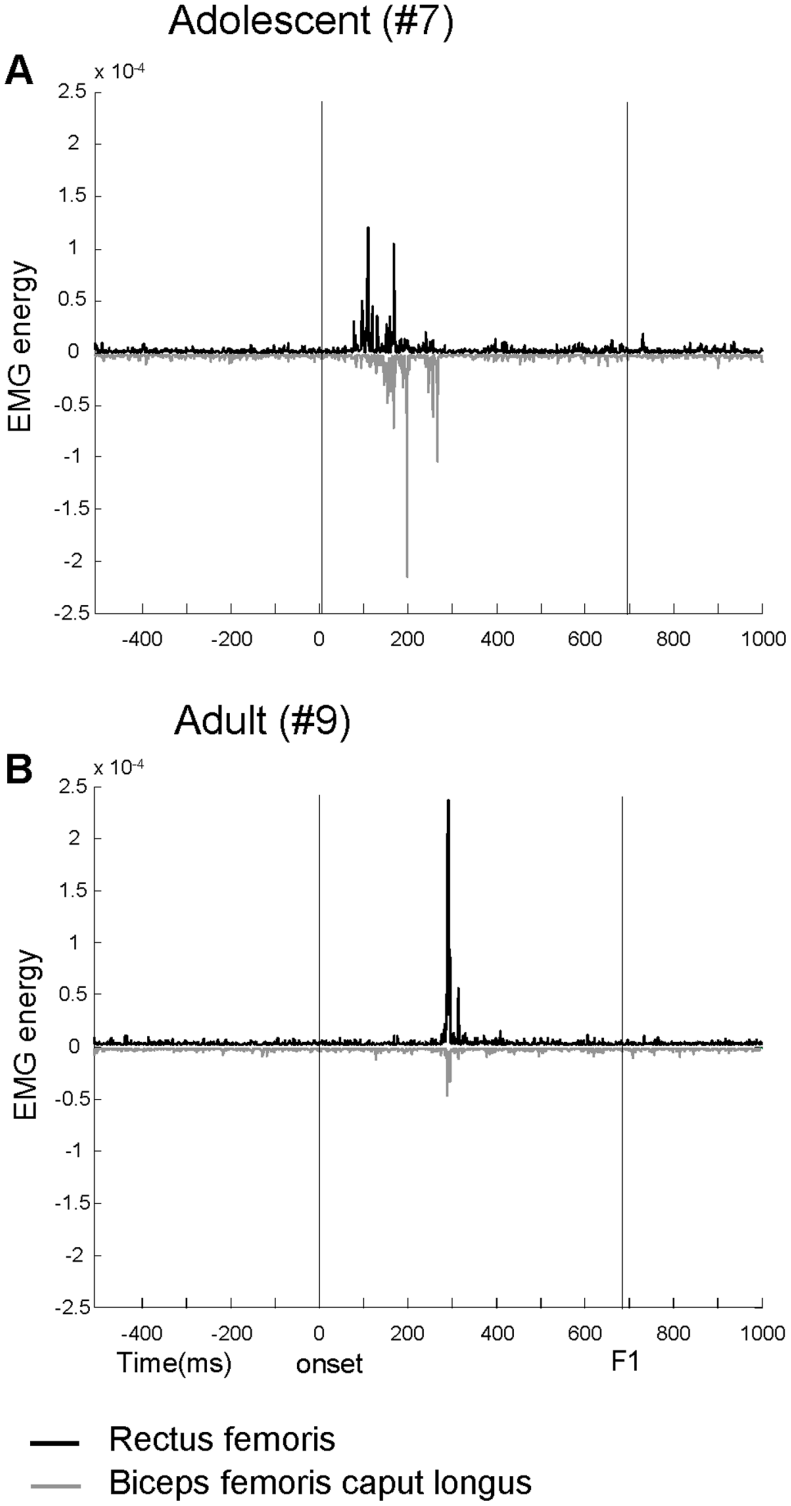
Examples of agonist-antagonist co-activation. Agonist-antagonist co-activation of Rectus femoris (black traces) and Biceps femoris (gray traces) recorded in one adolescent (A) and one adult (B) with Down syndrome. Vertical lines delimit the interval between the onset of the leg fall and the time of reversal at the end of the first flexion (F1). The traces represent the EMG with TKEO signal condition.

The activity of the RF was recorded in all of the subjects, but restricted to seven subjects we also recorded the EMG activity of BFcl muscle (see Materials and Methods). Figure 5A–B displays two examples of typical agonist-antagonist co-activation occurring during the first leg swing in four out of seven subjects. Although the timing of response from the two muscles was roughly synchronous, the pattern of EMG amplitude showed some variability over the trials recorded in the four subjects. For example, the traces from the adolescent represented in Fig. 5A showed a similar onset time, considering the criterion used for TKEO analysis (see Materials and Methods), but the first activation of BFcl raises with a much lower rate with respect the first burst exhibited by the RF. It is noteworthy that responses latency observed in these two subjects matched those detected in the subjects illustrated in Fig. 2A–B: in both cases the adults exhibited longer latencies than adolescents. In the remaining three subjects the BFcl displayed a tonic low activity throughout all the oscillations. Since the small sample of BFcl EMG traces and given that the phasic activity was absent or synergistic with that of the RF, for the further EMG analyses we considered only the activations of the RF muscle.

**Figure 6:**
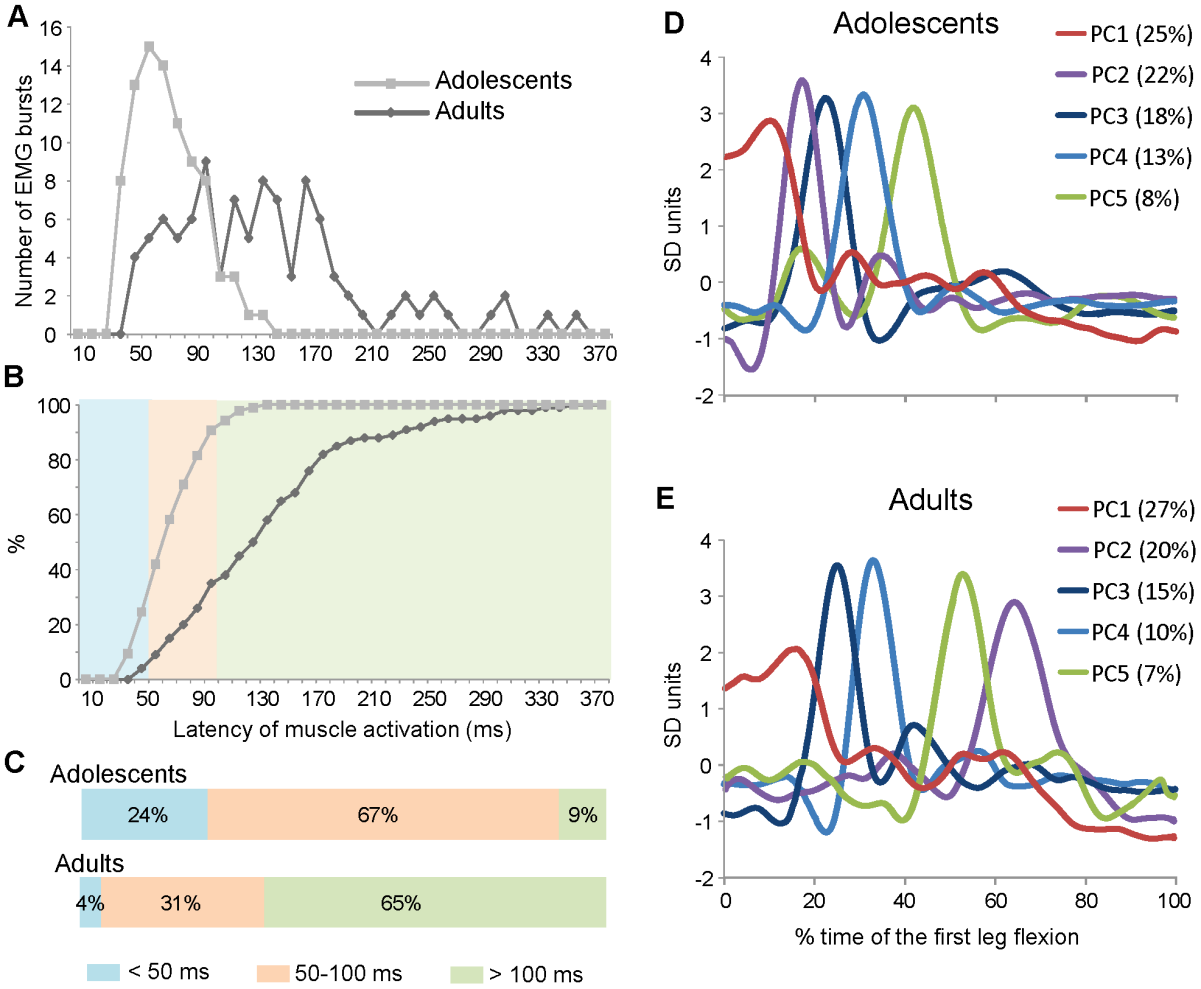
EMG latency distributions and Principal Components Analysis. Distribution of EMG latency (A–C) and principal components waveforms (D–E) over the interval of the first flexion. The EMG latency was measured from the onset of the leg fall and it is represented as distribution (A) and cumulative (B) frequency histograms. The latency distributions were parceled in three bands and expressed as fractions of the total measurements (C). Principal components analysis was performed on the two datasets of EMG responses recorded in adolescents (D) and in adults (E) with Down syndrome. The first five components accounted for 86% of the total variance in the adolescents and for 79% in the adults. For each component is reported the fraction of variance accounted. The time base is expressed as percentage of the first flexion length.

The most important differences between the two groups were detected in the temporal distribution of the RF bursts within the interval of the first flexion. Figure 6A–E illustrates the results obtained using trial-by-trial measurements of the latency of EMG responses (Fig. 6A–C) and by evaluating the distribution of the most common EMG waveforms as elaborated by the PCA (Fig. 6D–E).

The latency distribution profiles illustrated in figure 6A show that the two samples are significantly separated (p<0.001). Most of the latencies in the group of adolescents were concentrated in the region with the lowest values (67±23 ms; grey line), while the adults exhibited a broadly distribution over the first swing with a shift to the highest values (137±67 ms). The same data are represented as cumulative frequency plots in figure 6B. For a functional evaluation of these distributions we segregated the latencies within three time intervals (Fig. 6B–C): below 50 ms, compatible with short-latency reflexes (light blue band); between 50 and 100 ms, associated with long-latency reflexes (orange band); above 100 ms, associated with voluntary responses (green band). Most of the responses of adolescents occurred at short-latency (24%) or between 50 and 100 ms (67%), while almost all the latencies displayed by adults were within long-latency periods (31% between 50 and 100 ms; 65% over 100 ms).

We used the PCA to summarize the most common EMG waveforms exhibited by the subjects for each group (Fig. 6D–E). The first five PCs accounted for 86% and 79% of the total variance in adolescents and adults, respectively. Higher order PCs each explained small fraction of the variance (<5%) and described high-frequency oscillations of the EMG activity, hardly distinguishable from noise. In agreement with the results of the previous analysis, adolescents exhibited times of latency shorter than adults. The responses onset occurred over the first third of the first flexion for adolescents, with the temporal distribution of PCs waveforms directly related to the magnitude of variance explained (from PC1 to PC5; Fig. 6D). The adults showed all the waveforms delayed and distributed across the entire first half of the first swing; interestingly, the PC2, which accounted for 20% of the total variance (purple line in Fig. 6E), occurred later with respect to the other responses. Given the small size of our samples, we verified if the PCs waveforms were associated univocally to single subject or they can represent patterns of response distributed across the subjects included in each group. To this aim we determined the weighting coefficients for each PC, over the trials of the two datasets. The results of this analysis are reported in table 2 where the numbers represent the average values of the weighting coefficients from the trials of each subject. We set at 0.4 an arbitrary threshold value of weighting coefficients to highlight the level of contribution of each subject to the single PC waveforms (in bold in the table 2). Only one PC for each group exhibited a weighting coefficient above 0.4, associated to a single subject (PC3 in adolescents and PC4 in adults). More in general, as the fraction of variance explained extended, the number of subjects which contributed to each PC increased (4–6 subjects contributed to the PC1 or PC2 in both the groups). Therefore, most of the variance explained by the first five PCs represents modalities of EMG responses consistent across the subjects.

**Table 2:** Weighting coefficients computed for the first five Principal Components with respect to the original traces recorded in each adolescent and adult with Down syndrome.

Adolescents	#1	#2	#3	#4	#5	#6	#7	#8	#9	#10
PC1	0.23	0.04	0.16	**0.71**	**0.66**	0.11	**0.50**	0.27	**0.50**	**0.51**
PC2	**0.40**	**0.42**	0.34	0.21	0.23	**0.61**	0.15	**0.50**	0.22	**0.46**
PC3	0.11	0.25	**0.48**	0.11	0.18	0.27	0.09	0.14	0.13	0.26
PC4	0.23	0.11	0.20	0.38	0.38	0.24	**0.72**	**0.63**	**0.60**	0.31
PC5	0.09	**0.65**	**0.45**	0.17	0.24	0.28	0.12	0.22	0.22	0.26

Adults	#1	#2	#3	#4	#5	#6	#7	#8	#9	#10
PC1	0.10	**0.52**	**0.77**	**0.45**	0.20	0.15	**0.71**	0.17	**0.62**	**0.62**
PC2	**0.43**	**0.41**	0.16	0.31	0.23	**0.58**	0.21	**0.40**	0.14	0.14
PC3	0.14	0.09	0.19	0.12	**0.62**	0.07	0.18	**0.41**	0.29	0.17
PC4	**0.40**	0.17	0.20	0.19	0.16	0.06	0.16	0.39	0.16	0.24
PC5	0.35	0.30	0.22	**0.57**	0.28	**0.55**	0.28	**0.43**	0.39	0.24

PC, principal component. The absolute values of *r>*0.4 are in bold.

**Figure 7:**
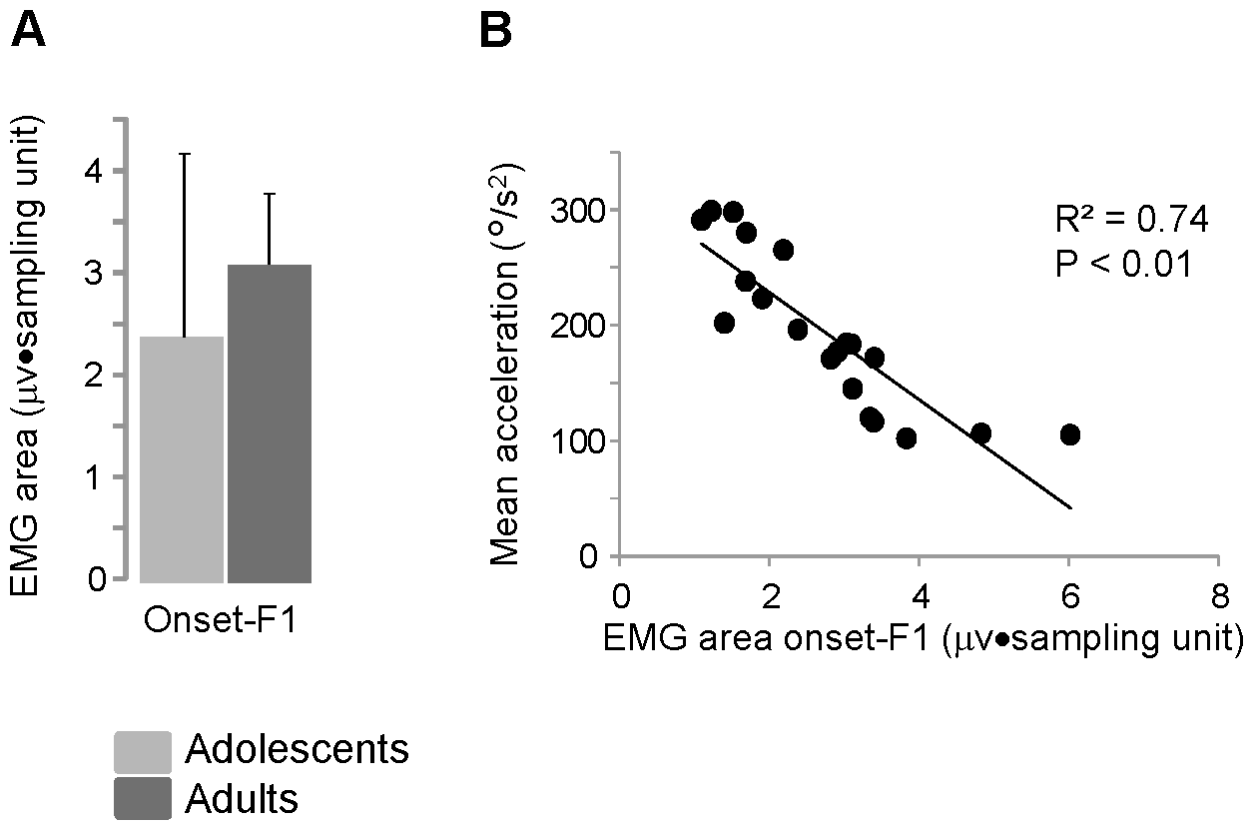
Quantification of the Rectus femoris activity and its relationship with movement acceleration. (A) Mean values and standard deviations of the total area of the Rectus femoris EMG activity recorded over the first flexion (from the onset of the leg fall to the first flexion peak, F1). (B) Relationship between changes of mean acceleration and EMG area during the first flexion. Each data point represents the average value obtained from 10 trials executing by each participant.

Regardless of the time of the burst occurrences, the amount of EMG activity, computed as EMG area over the first swing, showed no significant differences between the two groups (Fig. 7A). The level of muscle activation was the main contributor to explain the mechanical behavior observed across the subjects during the first flexion. In fact, collecting the data from all of the subjects and comparing the EMG area with the mean acceleration during the first flexion, the EMG activity accounted for 74% of the total variance associated with the changes of acceleration (Fig. 7B).

### Variability analysis

**Figure 8:**
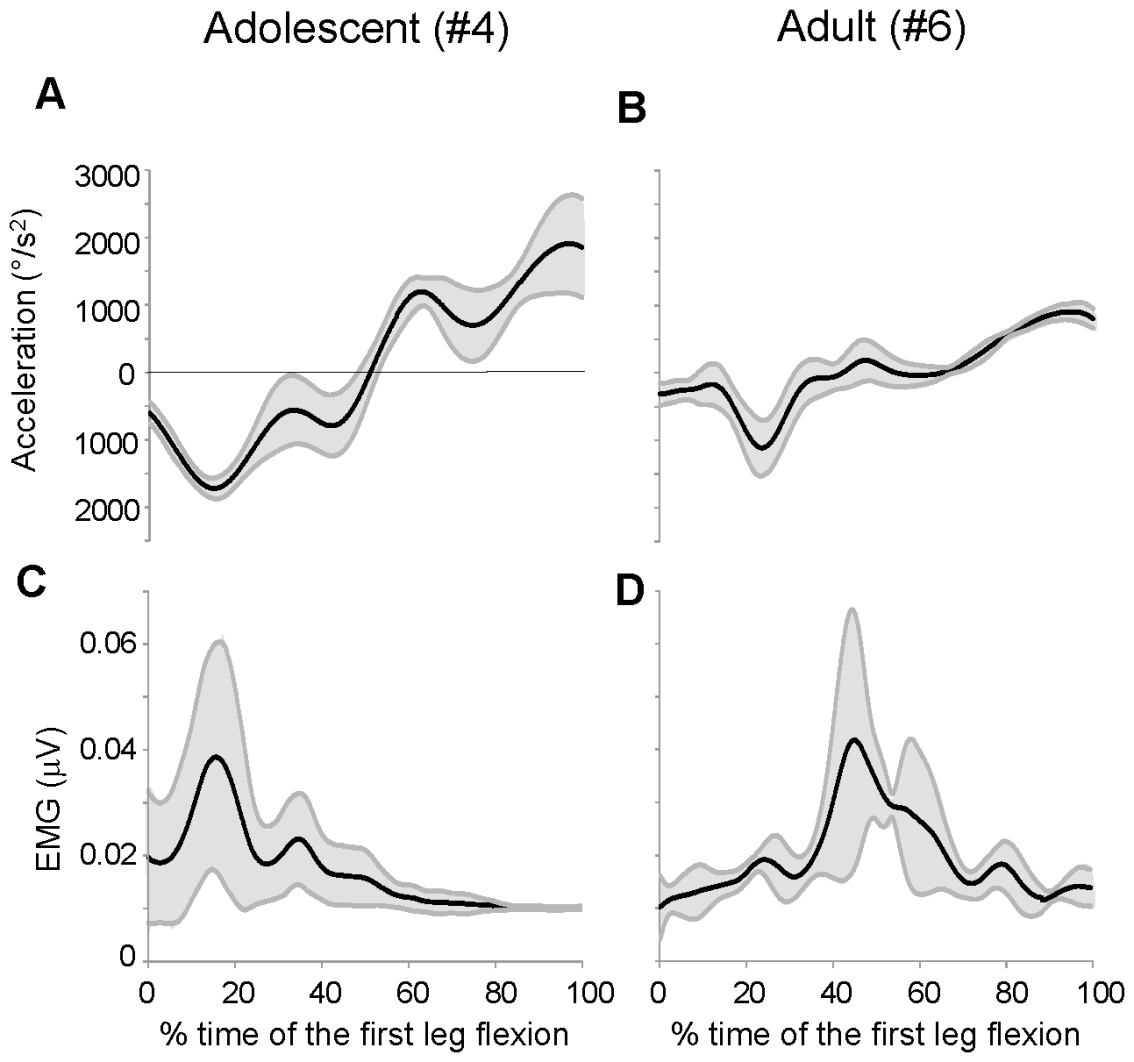
Examples of variability of kinematic and EMG traces. Averaged acceleration (A–B) and EMG (C–D) traces for one adolescent and one adult with Down syndrome over the interval of the first flexion. Each data point reports the mean value (black lines) and ±1 standard deviation (grey lines) computed from 10 trials. The time base is expressed as fraction of the first flexion length.

To find evidences for the effects of changes in EMG onset on the kinematics of the first swing, we computed the amount of movement variability over the acceleration profile and detected the times of occurrence of the minimum and the maximum variability. In figure 8A–D are illustrated two examples of temporal comparisons between acceleration profiles (Fig. 8A–B) and EMG activities (Fig. 8C–D): for each data point is reported the average value computed across 10 trials (black lines) and the associated variability expressed as ±1 standard deviation (grey lines). For both subjects, the timing of the minimum variability of acceleration changes in parallel with the timing of EMG onset. According to the delayed EMG activity described for the group of adults, in these examples the minimum kinematic variability was shifted later in the adult subject with respect to the adolescent. Therefore, the EMG bursts approximated the time of the maximum kinematic stability.

**Figure 9:**
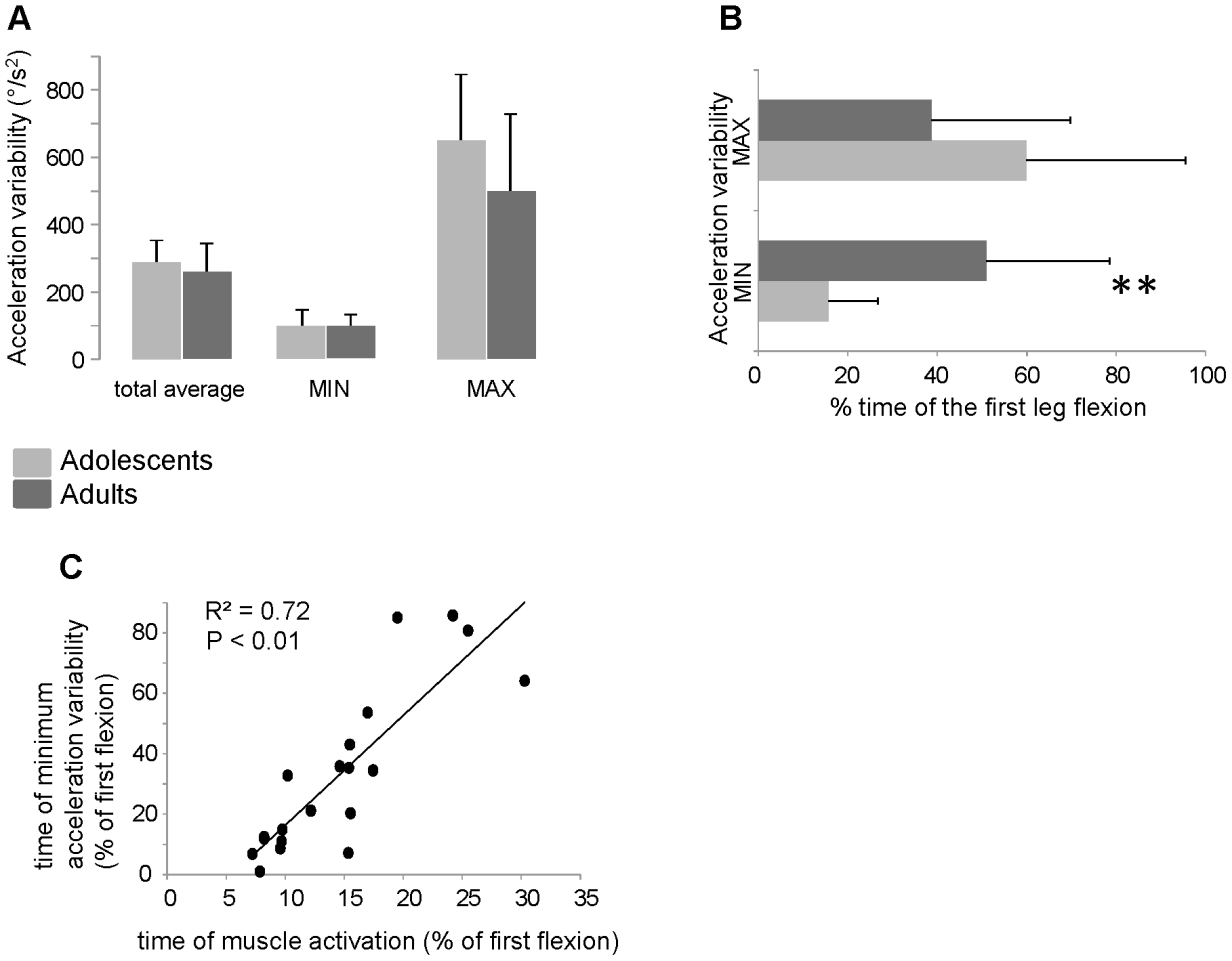
Analysis of kinematics variability and its relationship with muscle activation. (A) Means and standard deviations of the amount of acceleration variability. For each subject, the variability was estimated as the mean value from the standard deviations of all data point over the first flexion (total average) and from the standard deviations of the 10 trials at the time of the minimum (MIN) and maximum (MAX) variability. (B) Means and standard deviations of the timing of occurrence of the minimum and maximum variability expressed as fraction of the first flexion length. (C) Linear regression between the time of muscle activation and the time of minimum acceleration variability computed by the data from each subject (black circles). The axes values are fractions of the first flexion length. ** P<0.01.

The statistical analysis reveals that the time of occurrence of minimum variability changes regardless of the amount of the total variability over the interval of first flexion or at the time of minimum and maximum variability (Fig. 9A–B). In fact, although no significant difference was found for the quantity of acceleration variability between the two groups (Fig. 9A), important changes were detected in the time of minimum variability (Fig. 9B): for the adults, the minimum variability occurred at an averaged time corresponding to 51±27% of the total first flexion length, while for the adolescents the minimum variability was exhibited at 16±11% of the total time of the leg fall (p<0.001). The maximum variability showed a mirror behavior with an averaged time occurred at 60±36% and 39±31% of the total time respectively for adolescents and adults. However, in this case the difference was not significant (p = 0.17). To quantify the relationship between the timing of the minimum acceleration variability and the latency of the EMG bursts, we correlated the two parameters as fractions of the interval of the first flexion (Fig 9C). The linear regression shows a parallel increase of the time of the minimum variability and the latency of EMG activity with a good coefficient of determination (R^2^ = 0.72; p<0.01).

## Discussion

In a previous work we reported that adults with DS executing the Wartenberg test, reacted to the first leg fall producing bursts of activity in the RF [11]. In the current experiments we showed that adolescents with DS also exhibited similar behavior, but the muscle activation was significantly anticipated with respect to the adults. The timing of the muscle response was associated with the time of maximum kinematic stability over the interval of the first knee flexion. Thus, adolescents stabilized early the joint motion while adults delayed the time to maximize the stability of the leg fall. Generally, these activations are in line with the idea that, given the joint laxity and the muscle hypotonia inherent to DS, an appropriate pattern of muscle contraction is required to increase joint stability [9], [27], [28]. We believe that the data collected here and the associated elaborations may provide some insights for better understanding the biomechanical and neural adaptations underlying this behavior.

### Development and adaptability in Down syndrome

The results reported in this paper indicate that the temporal modulation of the muscle response changes during the development time. Usually, children with DS show motor development delayed with independent walking and upright posture achieved 1 year later compared to infants with typical development [19], [29], [30]. In preadolescents most of the basic motor abilities are developed but they continue to have difficulty in performing some motor skills [18], [31] and fail to increase muscle strength [32]. However, preadolescents and adolescents still have wide possibility to adapt and develop novel motor tasks [10], [21], [27], [33]. Smith et al. [21] argued that during the preadolescence, persons with DS express the best capability to select new patterns for motor adaptation. These authors evaluated the movement variability during gait in preadolescents ranged between 8–10 years, reporting that their gait was more adaptable than younger or older subjects (see also [27]). However, since in their study there were no participants aged between 11–35 years, it is not clear if such level of adaptability also exists in adolescents and young adults. Several works indicate that the effects of aging on motor performance in DS starting from about 35 years [10], [33]–[36]. Therefore, the data obtained by our samples, ranged between 9 and 31 years, can be enclosed in a framework where adaptive selection of new motor behaviors can be acquired and consolidated.

### Muscle activations to overcome joint laxity and hypotonia

The modulation of muscle reaction to an abrupt, passive leg flexion is one possible mechanism of adaptation implemented by persons with DS already in the childhood. To actively increase joint stiffness, they can employ either an agonist-antagonist co-contraction or a robust contraction of the antagonist muscle. Regardless the time of burst occurrence, we observed both the strategies across adolescents and adults. In general, ligament laxity and hypotonia decrease from toddlers to preadolescents, but these deficiencies accompany people with DS during lifespan [10], [33], [37]. Compensatory muscle activation patterns are accomplished especially when they faced with novel motor task or unexpected perturbations [6], [7], [38]. The possibility to shift back to a co-contraction after a pattern of reciprocal contraction has been acquired, may occur when a well learned task is perturbed. Ulrich et al. [27] and Smith et al. [8], demonstrated that, after learning to reduce stiffness during walking on the ground by coordinating reciprocal activation, preadolescents with DS restored patterns of co-contraction when they walked on a treadmill and the gait was perturbed by changing speed. Thus, persons with DS can modulate the patterns of muscle activation depending on the amount of practice and on the level of mechanical challenge. This flexibility in the muscle activation might explain a certain variability that we observed in the shape of the EMG co-activation. In fact, although the timing between the two muscles was comparable, large variations of EMG amplitude occurred in those subjects displaying muscle co-activation (see fig. 5).

The two patterns of activation, observed in seven participants, were independent of the group and could be associated to the different personal experiences lived by each subject: who has practiced a sufficient amount of alternating contractions, activated only the RF to increase stability immediately after the sudden fall of the leg; others, to maintain the proper leg stability, forced their muscles to perform co-activations.

During the oscillations following the first, subjects showed a basic muscle tone with a consistent level of activation. The parallel unchanged values of the damping coefficient observed across the interval between F1 and F4, supporting our earlier suggestion that damping coefficient might be a good predictor of muscle hypotonia in DS [11].

### Temporal changes in muscle activation may increase resource capabilities

The most important variation of muscle activity observed in the current study regarded the temporal changes in the EMG latency between adolescents and adults. In fact, the duration and the overall EMG area and the total amount of variance explained by the first five PCs during the first flexion, were similar between the two groups. The delayed timing of EMG activity observed in adults with respect to adolescents increased the movement stability at the end of the first flexion. This was validated by the reduction of variability at this time as well as by the reduction of amplitude of the second peak acceleration and by the increase of passive stiffness at the second flexion. We propose that this temporal shift of muscular activity might emerge from an adaptive process aimed to modulate the timing of movement variability rather than its total average level. By this modulation individuals with DS might select the time for muscle activation according to the need to increase the stability at a particular moment of the movement execution.

Movement variability is an inherent characteristic of the DS, explaining many of the motor deficiencies typical of this condition [10], [39]–[41]. The development process and the effects of the practice reduce movement uncertainty, but in several instances, the pattern of variability instead of the total amount of variability, contributes to the level of adaptation [20], [21], [42]. For example, Smith et al. [21] used nonlinear tools (see [42]) to analyze local variability during locomotion in individuals with DS and found that some parameters of the gait cycle were more instable and irregular in preadolescents than in toddlers or adults over 35 years. Since preadolescents showed also a better walking performance than their younger and older peers, these authors concluded that increasing variability enables preadolescents to access to a large number of actions and to select the behavior that best fits their needs. Similar conclusions were achieved by Black et al. [20], who quantified the movement variability by using the uncontrolled manifold analysis (see [43]). In this case preadolescents with DS used more adaptive strategies than controls to stabilize the center of mass and the head position at heel contact during locomotion. These examples would strengthen the possibility that the increase of EMG latency between adolescents and adults reported in our experiments, serves to provide more extensive limits to choose the time for an adaptive muscular activation. Unfortunately, the cross-sectional design of this study prevents us to clearly identify the age of transition from short to long latency selection of muscle activation, but it is realistic to think that the timing recorded in the adults may represent the final step of a process started during the early adolescence.

An alternative explanation for the latency delay observed in adults may be a deterioration of the short-reflex response associated with an anticipation of the aging effect. In this case, being the spinal reflex a quite fixed process, we would expect a more homogenous shift of the latency distribution from the adolescents to adults. Instead, the increased variability of the latency distribution in adults indicates an enrichment of the response flexibility (see Fig. 6). In addition, the excessive slowing of the spinal reflexes (from 67 ms to 137 ms in average) should produce serious impairments for most of basic motor tasks, contrasting many studies that indicate the age of about 35 years as the starting time for functional and behavioral deteriorations in persons with DS [27], [34]–[36]. Although an increasing of irregularity in the patellar tendon reflex was observed in adolescents with DS [44], to our knowledge no author dealt with the specific aging effect on the short- and long-latency reflexes in DS. The studies on elderly persons without DS report a worsening of the stretch reflex only for the long-latency loop with no significant effects on the short-latency response [45], [46]. Given the stability displayed by the spinal stretch reflex, deriving from its basic role over the movement control, we believe that a functional decline of short-latency responses is not very consistent with the behavior observed in the current study.

To provide a more specific functional interpretation to our results, we have to consider that the passive movements of the Wartenberg test are uncommon in the everyday repertoire. Thus, there is the possibility that the delayed muscle activation might have been transferred from temporal patterns implemented during the practice of more common tasks, such as walking or upright standing. Transfer from an experienced to a novel motor task was reported for persons with DS during single-joint elbow movements [7] or in manual actions [47]. The leg oscillatory movement during the swing phase of walking could be a good candidate for the origin of a pattern compatible with the pendular movements of the Wartenberg test. In fact, an increase of stiffness by specific muscle contractions occurs in persons with DS at the end of the swing phase to prevent the deficits in passive energy storage at the moment of foot impact [27], [48]. Such muscular activations appear in the early maturation of walking in DS toddlers [9]. Thus, the muscle responses observed in our study could origin from a process of generalization of temporal patterns of muscle activation elaborated to increase stiffness during gait [28], [49] or in upright posture [2], [50].

### Neuronal circuits underlying temporal changes in muscle activation

An efficient reaction to a sudden perturbation depends on a feedback control system where an error signal, carried by sensory afferences, is processed to restore a given set point. In our test, muscle spindles and joint receptors should be the main sources of sensory signals informing the brain on the onset of the leg fall. Spinal cord uses this information to elaborate very fast motor responses such as the monosynaptic stretch reflex, exhibiting latency of muscle activation below 50 ms. Later, the signal reaches cortical or subcortical structures producing long-latency reflexes that are non-voluntary feedback reactions with latencies between 50–100 ms. Above 100 ms, long-latency reflexes may be superimposed by voluntary feedforward activity [15]–[17], [51]. Almost all the latencies recorded in adults (97%) can be considered long-latency reflexes or voluntary responses while adolescents showed a preponderance of short- and long-latency reflexes (90%; see Fig. 6B–C).

When the time of the perturbation is unpredictable, like in the Wartenberg test, but the quantity can be noted (corresponding to the knee gravitation torque in our task), the nervous system may preprogram an appropriate level of muscle activation and wait for the sensory signal to drive the motor command. To this end are required high integrative circuits able to learn from previous experiences and transform the interaction between the limb and the environment into internal preprogrammed representations. Many studies demonstrated that, as long-loop circuits are involved, this process can take place, allowing the sensory signal to be more associated to the specificity of the task and the motor response to be more adaptive [17], [52]–[54].

If the shift from short to long-latency responses observed in this study between adolescents and adults depends on the formation of preprogrammed commands associated with long-latency reactions (as also suggested by other authors [6], [7], [38]), our expectation is that the nervous centers responsible for these responses, such as motor cortex, cerebellum and basal ganglia, should be functional in the DS. Numerous papers report that several parts of the brain of persons with DS exhibit diminished volumes, including frontal and temporal lobes, hippocampus and cerebellum [55], [56]. Most of these abnormalities may account for cognitive or emotional dysfunctions [1], but the motor cortices and the cerebellum can be more directly associated with motor impairments. Although cerebellum defects represent one of the more consistent neurological features in DS, conflicting relationship have been reported between this abnormality and motor dysfunctions [1], [57]–[59]. Furthermore, the modular organization of the cerebellum with several body maps distributed between cortex and deep nuclei [60], [61] provides a chance to persons with DS to implement preprogrammed reactions also in a small cerebellum. Another structure able to modulate reactive motor responses might be the basal ganglia which have normal dimensions in persons with DS [62]. It is noteworthy that no important morphological difference was found from the adolescence up to the age of 35–40 years [56], [63], indicating that motor adaptations occurring over these ages should be based on functional modification of the neural circuits.

Taken together, the above data suggest that the brain of persons with DS may have a good residual possibility to implement adaptive mechanical compensations, at least in the fashion of the feedback behavior.

### Conclusions

The changes in timing of muscle activation and the parallel reduction of joint movement variability observed in this study suggest that, between adolescence and adulthood, persons with DS explore a window of functional adaptations to optimize their inherent biomechanical deficits. According to the latency of muscle responses and to the possibility that the nervous system of persons with DS implements adaptive motor reactions, we believe that during the development time DS people are able to encode preprogrammed motor scheme to ever more improve the reactions to sudden perturbations.

Although our results represent generic signals to indicate the potentiality of adaptation in the persons with DS, we hope that they will help to motivate researchers and therapists to make every effort in discovering and fully stimulate the potential capacities of these persons not only during the early stage of motor development, but also over the lifetime. With these premises, Wartenberg test could be a valuable tool to investigate the basic motor adaptation in the DS and to provide an objective quantification for the unique biomechanics of people with this condition.

### Limitations of the study

We exclude the risk that the subjects have not understood the instructions to relax. In fact, in addition to our special care in providing clear guidelines, the consistency of EMG responses across trials and the constant low tonic activity during the oscillations following the first indicated that the intent of each subject was to achieve the maximum relaxation. When Wartenberg test was used in subjects with spasticity, they showed phasic muscle activations during the first drop and in the successive oscillations [13]. Moreover, since persons with DS are known for their slower reactions to a variety of stimulations [44], [64], we are confident that the bursts observed in these experiments depend on an existing adaptive mechanism.

This work has also the limit to be a cross-sectional study. However, we believe that the clarity of the differences between the two groups and the attempt for a comprehensive data quantification, can partially compensate for the benefit of a longitudinal design.

## References

[1] Lott IT, Dierssen M (2010) Cognitive deficits and associated neurological complications in individuals with Down's syndrome. Lancet Neurol 9: 623–633. 20494326 10.1016/S1474-4422(10)70112-5

[2] Shumway-Cook A, Woollacott MH (1985) Dynamics of postural control in the child with Down syndrome. Phys Ther 65: 1315–1322. 3162178 10.1093/ptj/65.9.1315

[3] Caselli MA, Cohen-Sobel E, Thompson J, Adler J, Gonzales L (1991) Biomechanical management of children and adolescents with Down syndrome. J Am Podiatr Med Assoc 81: 119–127. 1828503 10.7547/87507315-81-3-119

[4] Diamond LS, Lynne D, Sigman B (1981) Orthopedic disorders in patients with Down's syndrome. Orthop Clin North Am 12: 57–71. 6451852

[5] Cronk CE, Chumlea WC, Roche AF (1985) Assessment of overweight children with trisomy 21. Am J Ment Defic 89: 433–436. 3156498

[6] Latash ML, Almeida GL, Corcos DM (1993) Preprogrammed reactions in individuals with Down syndrome: the effects of instruction and predictability of the perturbation. Arch Phys Med Rehabil 74: 391–399. 8466421

[7] Almeida GL, Corcos DM, Latash ML (1994) Practice and transfer effects during fast single-joint elbow movements in individuals with Down syndrome. Phys Ther 74: 1000–1016. 7972361 10.1093/ptj/74.11.1000

[8] Smith BA, Kubo M, Black DP, Holt KG, Ulrich BD (2007) Effect of practice on a novel task-walking on a treadmill: preadolescents with and without Down syndrome. Phys Ther 87: 766–777. 17442836 10.2522/ptj.20060289

[9] Chang CL, Kubo M, Ulrich BD (2009) Emergence of neuromuscular patterns during walking in toddlers with typical development and with Down syndrome. Hum Mov Sci 28: 283–296. 19272665 10.1016/j.humov.2008.12.002

[10] Rigoldi C, Galli M, Albertini G (2011) Gait development during lifespan in subjects with Down syndrome. Res Dev Disabil 32: 158–163. 20943345 10.1016/j.ridd.2010.09.009

[11] Casabona A, Valle MS, Pisasale M, Pantò MR, Cioni M (2012) Functional assessments of the knee joint biomechanics by using pendulum test in adults with Down syndrome. J Appl Physiol 113: 1747–1755. 22995394 10.1152/japplphysiol.00960.2012 PMC3544505

[12] Wartenberg R (1951) Pendulousness of the legs as a diagnostic test. Neurology 1: 8–24. 10.1212/wnl.1.1.18 14785753

[13] Fowler EG, Nwigwe AI, Ho TW (2000) Sensitivity of the pendulum test for assessing spasticity in persons with cerebral palsy. Dev Med Child Neurol 42: 182–189. 10755458 10.1017/s0012162200000323

[14] Valle MS, Casabona A, Sgarlata R, Garozzo R, Vinci M, et al. (2006) The pendulum test as a tool to evaluate passive knee stiffness and viscosity of patients with rheumatoid arthritis. BMC Musculoskelet Disord 7: : 89. Available: http://www.biomedcentral.com/1471-2474/7/89. Accessed 02 July 2013. 10.1186/1471-2474-7-89 PMC1693559 17134492

[15] Marsden CD, Rothwell JC, Day BL (1983) Long-latency automatic responses to muscle stretch in man: Origin and function. Adv Neurol 39: 509–539. 6229160

[16] Matthews PB (1991) The human stretch reflex and the motor cortex. Trends Neurosci 14: 87–91. 1709536 10.1016/0166-2236(91)90064-2

[17] Pruszynski JA, Scott SH (2012) Optimal feedback control and the long-latency stretch response. Exp Brain Res 218: 341–359. 22370742 10.1007/s00221-012-3041-8

[18] van Gameren-Oosterom HB, Fekkes M, Buitendijk SE, Mohangoo AD, Bruil J, et al (2011) Development, problem behavior, and quality of life in a population based sample of eight-year-old children with Down syndrome. PLOS ONE 6: e21879 doi:10.1371/journal.pone.0021879. Accessed 02 July 2013 21814560 10.1371/journal.pone.0021879 PMC3140989

[19] Pereira K, Basso RP, Lindquist AR, da Silva LG, Tudella E (2013) Infants with Down syndrome: percentage and age for acquisition of gross motor skills. Res Dev Disabil 34: 894–901. 23291506 10.1016/j.ridd.2012.11.021

[20] Black DP, Smith BA, Wu J, Ulrich BD (2007) Uncontrolled manifold analysis of segmental angle variability during walking: preadolescents with and without Down syndrome. Exp Brain Res 183: 511–521. 17717659 10.1007/s00221-007-1066-1

[21] Smith BA, Stergiou N, Ulrich BD (2011) Patterns of gait variability across the lifespan in persons with and without down syndrome. J Neurol Phys Ther 35: 170–177. 22052133 10.1097/NPT.0b013e3182386de1 PMC3223537

[22] White H, Uhl TL, Augsburger S, Tylkowski C (2007) Reliability of the three-dimensional pendulum test for able-bodied children and children diagnosed with cerebral palsy. Gait Posture 26: 97–105. 16962781 10.1016/j.gaitpost.2006.07.012

[23] Li X, Zhou P, Aruin AS (2007) Teager-Kaiser energy operation of surface EMG improves muscle activity onset detection. Ann Biomed Eng 35: 1532–1538. 17473984 10.1007/s10439-007-9320-z

[24] Solnik S, Rider P, Steinweg K, DeVita P, Hortobágyi T (2010) Teager-Kaiser energy operator signal conditioning improves EMG onset detection. Eur J Appl Physiol 110: 489–498. 20526612 10.1007/s00421-010-1521-8 PMC2945630

[25] Glaser EM, Ruchkin DS (1976) Evoked potentials: Principal components and varimax analysis. In: New York Academic Press. Principles of neurobiological signal analysis. New York. pp. 233–285.

[26] Winter DA (2009) Anthropometry. In: John Wiley & Sons, editors. Biomechanics and motor control of human movement. Hoboken. pp. 82–106.

[27] Ulrich BD, Haehl V, Buzzi UH, Kubo M, Holt KG (2004) Modeling dynamic resource utilization in populations with unique constraints: preadolescents with and without Down syndrome. Hum Mov Sci 23: 133–156. 15474174 10.1016/j.humov.2004.06.002

[28] Galli M, Rigoldi C, Brunner R, Virji-Babul N, Giorgio A (2008) Joint stiffness and gait pattern evaluation in children with Down syndrome. Gait Posture 28: 502–506. 18455922 10.1016/j.gaitpost.2008.03.001

[29] Jobling A, Mon-Williams M (2000) Motor development in Down syndrome: a longitudinal perspective. In: Weeks DJ, Chua R, Elliott D, editors. Perceptual-motor behaviour in Down syndrome. Champaign, IL: Human Kinetics. pp. 225–248.

[30] Palisano RJ, Walter SD, Russell DJ, Rosenbaum PL, Gemus M, et al (2001) Gross motor function of children with Down syndrome: creation of motor growth curves. Arch Phys Med Rehabil 82: 494–500. 11295010 10.1053/apmr.2001.21956

[31] Connolly BH, Morgan SB, Russell FF, Fulliton WL (1993) A longitudinal study of children with Down syndrome who experienced early intervention programming. Phys Ther 73: 170–181. 8438005 10.1093/ptj/73.3.170

[32] Cioni M, Cocilovo A, Di Pasquale F, Araujo MB, Siqueira CR, et al (1994) Strength deficit of knee extensor muscles of individuals with Down syndrome from childhood to adolescence. Am J Ment Retard 99: 166–174. 7803033

[33] Rigoldi C, Galli M, Mainardi L, Crivellini M, Albertini G (2011) Postural control in children, teenagers and adults with Down syndrome. Res Dev Disabil 32: 170–175. 20933364 10.1016/j.ridd.2010.09.007

[34] Esbensen AJ, Seltzer MM, Krauss MW (2008) Stability and change in health, functional abilities, and behavior problems among adults with and without Down syndrome. Am J Ment Retard 113: 263–277. 18564887 10.1352/0895-8017(2008)113[263:SACIHF]2.0.CO;2 PMC2836825

[35] Dressler A, Perelli V, Feucht M, Bargagna S (2010) Adaptive behavior in Down syndrome: a cross-sectional study from childhood to adulthood. Wien Klin Wochenschr 122: 673–680. 21132392 10.1007/s00508-010-1504-0

[36] Carmeli E, Ariav C, Bar-Yossef T, Levy R, Imam B (2012) Movement skills of younger versus older adults with and without Down syndrome. Res Dev Disabil 33: 165–171. 22093661 10.1016/j.ridd.2011.09.008

[37] Parker AW, James B (1985) Age changes in the flexibility of Down's syndrome children. J Ment Defic Res 29: 207–218. 2933522 10.1111/j.1365-2788.1985.tb00330.x

[38] Latash ML, Corcos DM (1991) Kinematic and electromyographic characteristics of single-joint movements of individuals with Down syndrome. Am J Ment Retard 96: 189–201. 1834096

[39] Cioni M (2002) Gait analysis of individuals with Down syndrome. In: Esquenazi A, editor Physical Medicine and Rehabilitation, Gait Analysis, state of the art reviews. Philadelphia, PN: Hanley & Belfus. pp 303–323.

[40] Looper J, Wu J, Angulo Barroso R, Ulrich D, Ulrich BD (2006) Changes in step variability of new walkers with typical development and with Down syndrome. J Mot Behav 38: 367–372. 16968682 10.3200/JMBR.38.5.367-372 PMC2254170

[41] Agiovlasitis S, McCubbin JA, Yun J, Mpitsos G, Pavol MJ (2009) Effects of Down syndrome on three-dimensional motion during walking at different speeds. Gait Posture 30: 345–350. 19595593 10.1016/j.gaitpost.2009.06.003

[42] Stergiou N, Harbourne R, Cavanaugh J (2006) Optimal movement variability: a new theoretical perspective for neurologic physical therapy. J Neurol Phys Ther 30: 120–129. 17029655 10.1097/01.npt.0000281949.48193.d9

[43] Scholz JP, Schőner G (1999) The uncontrolled manifold concept: Identifying control variables for a functional task. Exp Brain Res 126: 289–306. 10382616 10.1007/s002210050738

[44] Morris AF, Vaughan SE, Vaccaro P (1982) Measurements of neuromuscular tone and strength in Down's syndrome children. J Ment Defic Res 26: 41–46. 6210779 10.1111/j.1365-2788.1982.tb00127.x

[45] Obata H, Kawashima N, Akai M, Nakazawa K, Ohtsuki T (2010) Age-related changes of the stretch reflex excitability in human ankle muscles. J Electromyogr Kinesiol 20: 55–60. 19303324 10.1016/j.jelekin.2009.01.009

[46] Klass M, Baudry S, Duchateau J (2011) Modulation of reflex responses in activated ankle dorsiflexors differs in healthy young and elderly subjects. Eur J Appl Physiol 111: 1909–1916. 21246214 10.1007/s00421-010-1815-x

[47] Edwards JM, Elliott D (1989) Asymmetries in intermanual transfer of training and motor overflow in adults with Down's syndrome and nonhandicapped children. J Clin Exp Neuropsychol 11: 959–966. 2531754 10.1080/01688638908400948

[48] Cioni M, Cocilovo A, Rossi F, Paci D, Valle MS (2001) Analysis of ankle kinetics during walking in individuals with Down syndrome. Am J Ment Retard 106: 470–478. 11531465 10.1352/0895-8017(2001)106<0470:AOAKDW>2.0.CO;2

[49] Gontijo AP, Mancini MC, Silva PL, Chagas PS, Sampaio RF, et al (2008) Changes in lower limb co-contraction and stiffness by toddlers with Down syndrome and toddlers with typical development during the acquisition of independent gait. Hum Mov Sci 27: 610–621. 18649965 10.1016/j.humov.2008.01.003

[50] Carvalho RL, Almeida GL (2009) Assessment of postural adjustments in persons with intellectual disability during balance on the seesaw. J Intellect Disabil Res 53: 389–395. 19143905 10.1111/j.1365-2788.2008.01147.x

[51] Petersen N, Christensen LO, Morita H, Sinkjaer T, Nielsen J (1998) Evidence that a transcortical pathway contributes to stretch reflexes in the tibialis anterior muscle in man. J Physiol 512: 267–276. 9729635 10.1111/j.1469-7793.1998.267bf.x PMC2231172

[52] Lewis GN, MacKinnon CD, Perreault EJ (2006) The effect of task instruction on the excitability of spinal and supraspinal reflex pathways projecting to the biceps muscle. Exp Brain Res 174: 413–425. 16676166 10.1007/s00221-006-0475-x PMC2756617

[53] Kurtzer IL, Pruszynski JA, Scott SH (2008) Long-latency reflexes of the human arm reflect an internal model of limb dynamics. Curr Biol 18: 449–453. 18356051 10.1016/j.cub.2008.02.053

[54] Pruszynski JA, Kurtzer I, Nashed JY, Omrani M, Brouwer B, et al (2011) Primary motor cortex underlies multi-joint integration for fast feedback control. Nature 478: 387–390. 21964335 10.1038/nature10436 PMC4974074

[55] Raz N, Torres IJ, Briggs SD, Spencer WD, Thornton AE, et al (1995) Selective neuroanatomic abnormalities in Down's syndrome and their cognitive correlates: evidence from MRI morphometry. Neurology 45: 356–366. 7854539 10.1212/wnl.45.2.356

[56] Pinter JD, Eliez S, Schmitt JE, Capone GT, Reiss AL (2001) Neuroanatomy of Down's syndrome: a high-resolution MRI study. Am J Psychiatry 158: 1659–1665. 11578999 10.1176/appi.ajp.158.10.1659

[57] Aylward EH, Habbak R, Warren AC, Pulsifer MB, Barta PE, et al (1997) Cerebellar volume in adults with Down syndrome. Arch Neurol 54: 209–212. 9041863 10.1001/archneur.1997.00550140077016

[58] Hyde LA, Crnic LS, Pollock A, Bickford PC (2001) Motor learning in Ts65Dn mice, a model for Down syndrome. Dev Psychobiol 38: 33–45. 11150059 10.1002/1098-2302(2001)38:1<33::aid-dev3>3.0.co;2-0

[59] Galante M, Jani H, Vanes L, Daniel H, Fisher EM, et al (2009) Impairments in motor coordination without major changes in cerebellar plasticity in the Tc1 mouse model of Down syndrome. Hum Mol Genet 18: 1449–1463. 19181682 10.1093/hmg/ddp055 PMC2664148

[60] Manni E, Petrosini L (2004) A century of cerebellar somatotopy: a debated representation. Nat Rev Neurosci 5: 241–249. 14976523 10.1038/nrn1347

[61] Casabona A, Bosco G, Perciavalle V, Valle MS (2010) Processing of limb kinematics in the interpositus nucleus. Cerebellum 9: 103–110. 20013085 10.1007/s12311-009-0149-x

[62] Aylward EH, Li Q, Habbak QR, Warren A, Pulsifer MB, et al (1997) Basal ganglia volume in adults with Down syndrome. Psychiatry Res 74: 73–82. 9204510 10.1016/s0925-4927(97)00011-5

[63] Lott IT (2012) Neurological phenotypes for Down syndrome across the life span. Prog Brain Res 197: 101–121. 22541290 10.1016/B978-0-444-54299-1.00006-6 PMC3417824

[64] Lam MY, Hodges NJ, Virji-Babul N, Latash ML (2009) Evidence for slowing as a function of index of difficulty in young adults with Down syndrome. Am J Intellect Dev Disabil 114: 411–426. 19792057 10.1352/1944-7558-114.6.411

